# Clinical and immunological assessment of *Mycobacterium vaccae* (SRL172) with chemotherapy in patients with malignant mesothelioma

**DOI:** 10.1038/sj.bjc.6600063

**Published:** 2002-02-01

**Authors:** R Mendes, M E R O'Brien, A Mitra, A Norton, R K Gregory, A R Padhani, K V Bromelow, A R Winkley, S Ashley, I E Smith, B E Souberbielle

**Affiliations:** Lung Unit, Department of Haematology The Royal Marsden Hospital NHS Trust, Downs Road, Sutton, SM2 5PT, UK; Department of Molecular Medicine, The Rayne Institute, Kings College London, London, UK; Kent Cancer Center, Maidstone, Kent, ME16 9QQ, UK

**Keywords:** intra-pleural, chemotherapy, intradermal, vaccine, immunotherapy

## Abstract

The objectives of this study were to determine the toxicity of intratumoural/intrapleural SRL172 in addition to intradermal SRL172 and standard chemotherapy (mitomycin-C, vinblastine and cisplatin) in patients with malignant mesothelioma. Patients received chemotherapy (mitomycin-C: 8 mg m^−2^, vinblastine: 6 mg m^−2^, cisplatin 50 mg m^−2^) on a 3-weekly basis for up to six courses. IP SRL172 injections were given 3-weekly prior to chemotherapy and escalated in groups of three patients from 1 μg to 1 mg bacilli in 10-fold increments. Patients were also given ID SRL172 at a dose of 1 mg bacilli 4-weekly. Patients were assessed for toxicity after each course of chemotherapy and for response by CT imaging. Immuno-haematological parameters were analyzed pre-treatment and 1 month after completion of treatment. There was no dose limiting toxicity with IP SRL172 although there was greater toxicity at the highest dose (*n*=13). There were six out of 16 partial responses (37.5%). Haemato-immunological parameters, measured in seven patients pre and post-therapy, revealed that response rate correlated with a decrease in platelet count and there was an increase in activation of natural killer cells and a decrease in the percentage of IL-4 producing T cells in all tested patients post-treatment. SRL172 can be given safely into tumour deposits and the pleural cavity in patients with malignant mesothelioma and we have established the dose for phase II testing.

*British Journal of Cancer* (2002) **86**, 336–341. DOI: 10.1038/sj/bjc/6600063 www.bjcancer.com

© 2002 The Cancer Research Campaign

## 

The incidence of mesothelioma is rising, from around 1000 cases per year in the UK at present, to a predicted peak of nearly 3000 by the year 2020, i.e. 1% of all male deaths (Peto *et al*, 1995). This increase is due to asbestos exposure which continued into the late 1960s. The median survival from the time of diagnosis is 4–18 months with 5% of patients alive at 5 years. The factors which predict for poor outcome are male sex, performance status, non epithelial pathology, low haemoglobin, high white cell count and high platelet count (Curran *et al*, 1998; Herndon *et al*, 1998; Edwards *et al*, 2000). No single treatment modality appears to make a significant difference to the outcome of this disease (Ong and Vogelzang, 1996). There is therefore a great need for improved treatment in mesothelioma. Currently we use a combination chemotherapy, mitomycin, cisplatin and vinblastine (MVP), which has been shown to lead to symptomatic control and acceptable toxicity in patients with malignant mesothelioma (Middleton *et al*, 1998). With this chemotherapy regimen, we expect objective response rates of around 20%.

SRL172 is a suspension of heat-killed *Mycobacterium vaccae*, a fast growing avirulent mycobacterium, that may have non-specific immunomodulating properties, e.g. it may promote Th1 and down regulate Th2 cytokine production (Grange *et al*, 1995). The proposed rationale behind the combination was that the chemotherapy, although slightly immunosuppressive, could cause the *in vivo* release and subsequent presentation of tumour antigens and trigger a tumour specific response by the immune system when boosted by an immuno-modulator like SRL172. Previously we have investigated the role of intradermal SRL172 (4-weekly) in combination with MVP chemotherapy (3-weekly) in a randomized phase II trial of 28 symptomatic patients with non-small cell lung cancer (NSCLC) and mesothelioma. There was a trend towards improved response rate (54% *vs* 33%), improved median survival (9.7 months *vs* 7.5 months) and improved 1 year survival (42% *vs* 18%) in patients who received both chemotherapy and SRL172 compared to those in the chemotherapy alone group. When the data were analyzed separately for NSCLC and for mesothelioma, the potential benefit of the combination was seen in both cancers (O'Brien *et al*, 2000). We have also reported similar results in SCLC (Assersohn *et al*, 2002).

In this study, intrapleural/intratumoural SRL172 injections were added to intradermal SRL172 and chemotherapy in patients with malignant mesothelioma. The rationale behind this new study was that SRL172 in the tumour or in the pleural cavity could result in an increased concentration of the immunologically active compound in the vicinity of the tumour, thus causing an inflammatory reaction which could potentiate the anti-tumour killing at the tumour site. Indeed, it has been reported that the local tumour environment of malignant mesothelioma is immunologically suppressive, due to the secretion of cytokines such as TGF-β (Fitzpatrick *et al*, 1995; Marzo *et al*, 1997). Therefore changing this local immuno-suppressive environment to a more immuno-stimulating environment may be beneficial. SRL172 had not been administered previously in the pleural cavity, and thus we initiated a phase I/II clinical trial to document the safety and clinical efficacy of repeated injections of intratumoral/intrapleural SRL172 in patients, with doses ranging from 1 μg to 1 mg of *M. vaccae* bacilli.

## PATIENTS AND METHODS

### Selection of patients

Intrapleural (IP) and intradermal (ID) SRL172 and chemotherapy were offered to symptomatic patients of performance status 2 and above with either a histological or cytological diagnosis of mesothelioma. Patients were recruited in four cohorts corresponding to increasing doses of IP SRL172. Baseline scans were analyzed and a site for a single IP SRL172 injection was chosen aiming for the greatest area of bulk noting the proximity of vital structures and depth of the chest wall at that point. If the disease was complicated by a large pleural effusion, this was drained prior to insertion of SRL172 into the cavity.

### SRL172 suspension

SRL172 was formulated as a suspension of 10 mg ml^−1^ heat-killed (autoclaved at 121°C for 15 min) *Mycobacterium vaccae* in borate buffered saline (pH 8) and was provided in 3 ml glass vials at a concentration of 10^9^ bacilli per 0.1 ml dose (1 mg) and stored refrigerated in the dark at 4°C in the hospital pharmacy until use.

### Treatment schedule

All patients received 3-weekly chemotherapy consisting of mitomycin-C (8 mg m^−2^, omitted courses 3 and 5), vinblastine 6 mg m^−2^ (maximum 10 mg) and cisplatin 50 mg m^−2^. All patients also received an intradermal injection of SRL172 (1 mg bacilli) 4-weekly, starting at the same time as the chemotherapy. SRL172 IP injections were given 3-weekly. The IP dose of SRL172 increased tenfold with each cohort vaccine starting at 1 μg bacilli (cohort 1), to 1 mg bacilli in the last cohort (cohort 4). The first IP SRL172 injection was given into the site of bulkiest disease or into the pleural cavity, at the same time as the first chemotherapy and the first intradermal injection of SRL172. Only when two patients had safely received the SRL172 for two courses could the next cohort commence treatment. Dexamethasone was omitted as an antiemetic during the first course of chemotherapy.

### Assessment of toxicity, objective and symptomatic response

Toxicity was assessed after each cycle of chemotherapy using CTC toxicity grading. Symptomatic responses and objective responses by means of chest X-ray, haematological and non-haematological toxicity were recorded 3-weekly. CT scans were repeated after three courses of chemotherapy and at the end of treatment.

Clinical outcome was defined as partial response (PR), minor response (MR), no change (NC) and progressive disease (PD). Partial response was defined as a decrease of 50% or more in the product of two diameters which is maintained for at least 1 month according to WHO criteria, a minor response was 25–49% shrinkage, and progressive disease was a 25% increase. The responses were also re-evaluated using the RECIST criteria (Therasse *et al*, 2000) at the end of the study which did not result in a change of response assessment.

Symptomatic response was defined as complete response (SCR) if the symptom disappeared, partial response (SPR) if the symptom improved, no change (SNC) and progressive disease (SPD) if the symptom did not change or became worse respectively.

### Immunological assays

Seven patients had blood taken at baseline and a repeat blood was taken 1 month after the end of treatment for immunological studies. As part of the protocol, for each patient, baseline blood was also taken from the spouse and from an age-sex-matched control. Paired blood samples were not taken in nine patients because they had either received prior chemotherapy, they refused to have blood taken for the immunological study, there was no suitable available control(s) at the time or they died before the end of the treatment. All assays were performed with whole blood requiring only 3.5 ml of blood per patient. Full blood count and differential counts were performed in a Coulter STKS machine in the Department of Haematology. Three colour Facscan analysis of lymphocytes markers were performed as described (Bromelow *et al*, 1998) and covered both T cell and NK markers. Mitogenic lymphocyte stimulations with PHA (10 μg ml^−1^, Murex), Pokeweed mitogen (10 μg ml^−1^, Sigma, Poole, UK) and SEB (10 μg ml^−1^, Sigma) were performed with 20 μl of blood diluted 1/10 RPMI1640 after 3 days incubation. Recall antigen stimulations with tetanus toxoid (5 μg ml^−1^, Connaught), Influenza virus (X31, 100 HAU ml^−1^, gift from Dr Brian Thomas, NIMR, Mill Hill, UK), tuberculin, (T147S strain, 5 μg ml^−1^, kind gift of Prof John Stanford, UCL, London, UK), *Mycobacterium vaccae* (25 μg ml^−1^, gift from J Stanford) and *Mycobacterium flavescin* (5 μg ml^−1^, gift from J Stanford) were performed in the same way as the mitogenic stimulations but with 5 days incubation. Optimum concentration for the mitogens, the recall antigens and the allogeneic stimulators had previously been determined in a pilot study with blood from normal individuals. Mixed lymphocyte reaction (MLR) was assayed using a pool of allogeneic PBMC prepared from 20 different individuals as described (Bromelow *et al*, 2001). Intracellular cytokine assays (IL-2, IL-4, γ-INF and TNF-α) were performed as described for both T cells (Jason and Larned, 1997) and NK cells (Mendes *et al*, 2000).

### Ethical considerations

The study was approved by the Research Ethics Committee at the Royal Marsden Hospital, Sutton, UK, and written informed consent was obtained from all patients.

## RESULTS

### Recruitment analysis

In total, 16 patients were recruited, mean age 62 years, range 53–73 years; 12 males, four females. Epithelial mesothelioma was the most common histological subgroup and 13 patients had a residual pleural effusion prior to chemotherapy, six of these patients had had a talc pleurodesis (Table 1

**Table 1:**
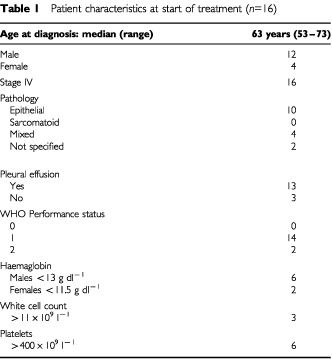
Patient characteristics at start of treatment (*n*=16)

). The haematological parameters of a raised white cell count, raised platelet count and low haemaglobin are included to describe the group as these are known poor prognostic factors and were present in up to 50% of this study group. Three patients had previously received cisplatin based chemotherapy. Table 2

**Table 2:**
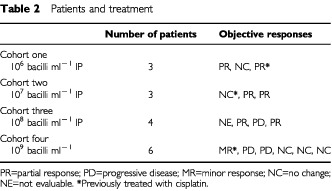
Patients and treatment

shows the patient numbers with the doses of intrapleural vaccine received and their overall responses to the treatment. The protocol recommended three patients to be tested in each cohort to make up 12 patients in total. However, four additional patients were recruited in this phase I study as per protocol to replace specific patients for the following reasons: three patients died within the first 3 weeks of the study, one from a myocardial infarction (cohort 3) and two patients (both from cohort 4) died of progressive disease. An extra patient was recruited to replace one patient (cohort 4), whose IP dose was reduced due to fever.

In total, 16 patients entered the trial, of which 13 patients were assessable for toxicity (two early deaths and one loss to follow-up did not have toxicity data documented). The median number of courses given was 3.5; 6 patients received six courses, two patients four courses, five patients three courses and the others ⩽two courses.

### Toxicity

All toxicity was recorded and graded according to the CTC toxicity guidelines every 3 weeks after chemotherapy. The main side effects were nausea and vomiting, constipation and hair loss but these were not severe. The incidence and grade of haematological toxicity was acceptable (Table 3

**Table 3:**
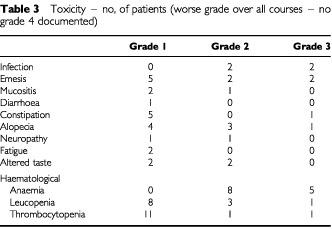
Toxicity – no, of patients (worse grade over all courses – no grade 4 documented)

). Five patients developed pain at the site of the ID SRL172 injection which was grade 1 and did not require treatment. Two patients reported grade 2 flu-like symptoms which resolved over 24 h, one of whom was admitted for observation. One of these patients had repeated fever with the intrapleural injections and thus the dose of intrapleural SRL172 was reduced with no further fever at the lower dose.

### Objective and symptomatic responses and survival

Overall, nine out of 16 patients (56%) reported symptomatic benefit from the treatment (Table 4

**Table 4:**
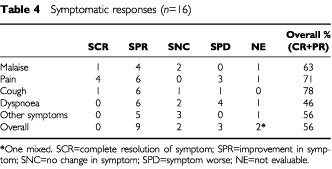
Symptomatic responses (*n*=16)

). Of the 14 patients with pain as a symptom, 10 patients reported improvement, and of the nine patients with cough, seven patients also described improvement. The most difficult symptom to treat in this series was dyspnoea with a response rate of 6 out of 13. Figure 1

**Figure 1:**
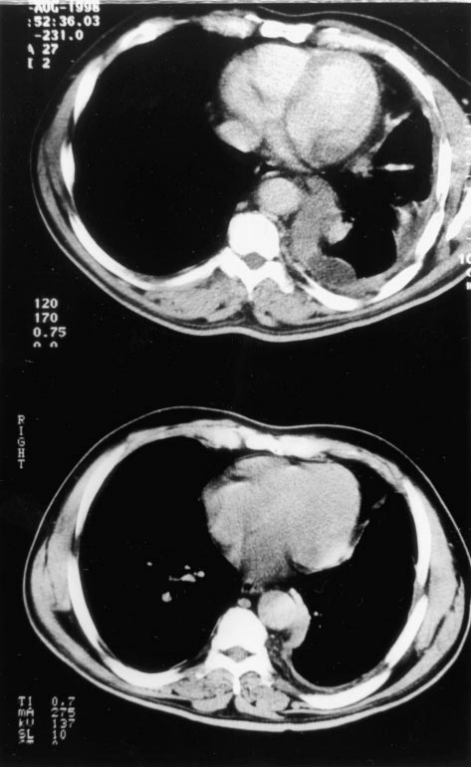
Pre- and post-treatment CT scan of responding patient.

illustrates the objective responses in one of the patients. Overall, there were six out of 16 partial responses (37.5%). For the first three cohorts there were responses in six out of nine assessable patients (one early death) including a PR in one patient who had been previously treated and responded to cisplatin. There was a MR in a second previously treated patient in cohort 4. There was no PD documented in the first two cohorts. There were more objective responses at the lower doses than at the higher doses (Table 2). The median overall survival was 10.5 months and disease free progression was 8 months (Figure 2

**Figure 2:**
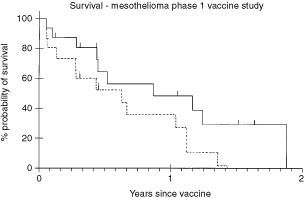
Survival (continuous line) and Progression-Free Survival (dashed line) for all patients.

).

### Immunological studies

**Figure 3:**
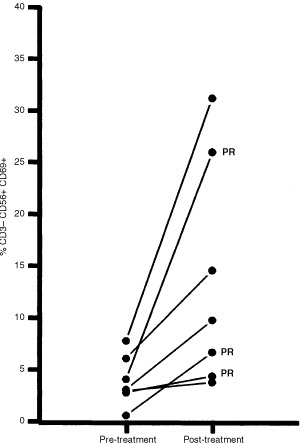
Percentage of peripheral blood CD3−CD56+ CD69+ activated NK cells (*y* axis) among gated lymphocytes in tested patients (*n*=9), pre-treatment and post-treatment (*x*-axis). Patients with partial response (PR) are highlighted.

**Figure 4:**
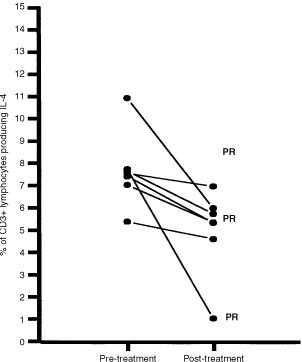
Percentage of peripheral blood CD3+ T lymphocytes producing IL-4 (*y* axis) among gated lymphocytes in tested patients (*n*=9), pre-treatment and post-treatment (*x*-axis). Patients with partial response (PR) are highlighted.

All haemato-immunological parameters tested were compared pre-and post-treatment with the view to correlating specific biological parameters with either response or treatment. Only one haematological parameter correlated with response; the platelet count decreased with treatment but the number of patients was too small to allow meaningful statistical analysis. When the immunological parameters were analyzed, there was no statistical difference pre-and post treatment for all types of lymphocytes proliferation (mitogenic, recall antigen and allogeneic stimulation), the T cell markers as well as for the production of cytokines by T cells and NK cells (data not shown). Only two parameters changed statistically with treatment in all patients tested; there was a significant increase in the percentage of NK cell activated as assessed by expression of CD69 on NK cells (CD3-CD56+ve gated lymphocytes) (Figure 3

); the mean value was 3.8%, standard deviation (s.d.±2.3) pre-treatment, and 13.7% (s.d.±10%) after treatment (paired *t*-test *P*=0.02, 2-tailed *P* value). The second observation was a decrease in the percentage of CD3+ T lymphocytes producing IL-4 after treatment (Figure 4

); the mean value was 7.6% (s.d.±1.6) pre-treatment, and 4.9% (s.d.±1.8%) after treatment (paired *t-*test *P*=0.02, 2-tailed *P* value).

## DISCUSSION

The patients selected for this study all had stage IV mesothelioma and symptoms from their disease were considered to be either a similar or worse group to our original report in which a response rate of 20% was obtained in a group of 39 chemotherapy naïve patients (Middleton *et al*, 1998). The median survival of the earlier group of patients was 6 months compared to 10.5 months in the current series which also included three patients who had previously received cisplatin based chemotherapy. Most of our patients were male, half of our patients had a low haemoglobin and six out of 16 had a raised platelet count. Of the other known prognostic factors most of our patients had the better prognostic epithelial histological subgroup, were of reasonable performance status (WHO 1) and only three had a high white cell count (Curran *et al*, 1998; Herndon *et al*, 1998; Edwards *et al*, 2000).

The good symptomatic response was as previously reported for the common symptoms with the exception of an improvement in ‘malaise’ – 39% response in the previous series and 63% in the current series – despite the omission of dexamethasone as an anti-emetic in most cases with chemotherapy. The higher than expected number of partial responses seen (six out of nine) in the first three cohorts of this study, with toxicity similar to that seen with chemotherapy suggests that there may be an advantage to the addition of local treatment to systemic treatment in the management of this disease.

We found that most patients after chemotherapy had a decrease in platelet count, however five out of six of the patients who had a documented PR by either WHO or RECIST criteria had at least a 30% decrease in the platelet count 1 month after their final chemotherapy compared to none out of three of patients with PD. The numbers are small and therefore the platelet count has to be formally evaluated in future large studies as a marker of this nature would have great practical implications in the assessment of response to treatment in this disease in which CT scan assessment is both difficult and resource intensive. Interleukin 6 has been implicated as one of the possible soluble factors responsible for the increase in platelet count seen in mesothelioma patients (Nakano *et al*, 1998). It is possible that treatment decreases interleukin 6 produced by the mesothelioma with a resulting decrease in the platelet count. As previously shown, high initial platelet load is an adverse prognostic factor in this disease (Herndon *et al*, 1998).

Combinations of chemotherapy and immunotherapy are not new in the treatment of mesothelioma. Cisplatin used with subcutaneous α-interferon at two different doses (3×10^6^ IU and 6×10^6^ IU) gave a response rate of 40 and 27% in 26 and 30 patients respectively but with significant haematological and non-haematological toxicity (Soulie *et al*, 1996; Trandafir *et al*, 1997). A reduced dose of cisplatin with mitomycin-C and α-interferon gave a response rate of 21% in 24 patients (Rodier *et al*, 1996).

The immunological testing in this study was essentially to screen different immunological parameters which may have been modified by the treatment and could in future studies lead to clarification of the mechanisms of action of the regimen. At this stage, we only included immunological parameters of T cell immunity (both Th1 and Th2 arms) and parameters of adaptive immunity (NK cells) following on from the hypothesis that SRL172 could be a non-specific immune modulator. SRL172 has been reported to increase interleulin 2 (IL-2) production in peripheral blood monocytes in patients with melanoma (Maraveyas *et al*, 1999) and we had also demonstrated that it leads to NK activation *in vitro* (KV Bromelow *et al*, in preparation). The immunological testing was only performed in the peripheral blood and future studies looking at possible immunological changes in the tumour are warranted. Local stimulation in itself may have a role in the treatment of malignant mesothelioma as in general this disease is localized for most of its history. We were unable to demonstrate increased IL-2 production by peripheral T cells (Th1) in this population of patients following treatment but local (intratumoural) production of cytokines is a possibility which should be tested in the future. IL-2 has been given into the pleura of patients with mesothelioma with some activity (Eggermont *et al*, 1991). The responses were seen at the lower dose level and IL-2 and tumour necrosis factor were not detectable in the serum. On the other hand, a significant decrease in the proportion of IL-4 producing CD3^+^ T cells (Th2) as well as an increase in NK activation was recorded in the peripheral blood after SRL172 and chemotherapy irrespective of the clinical response. At this stage we cannot say if these measurable biological modifications are due to SRL172, chemotherapy or both. However, these changes may have some direct correlation with response as it is possible that only a proportion of tumours are susceptible, for example, to NK cells (in the same way only a proportion of patients respond to chemotherapy despite all receiving the chemotherapy). Therefore possible tumour infiltration by NK cells and cytokine-producing CD3^+^ cells need to be studied in patients with mesothelioma who receive the regimen tested in this study.

The past 10 years have seen the arrival of a number of new drugs for the treatment of a number of solid tumours and in particular non-small cell lung cancer. Many of these new agents have now been tested in the treatment of mesothelioma both as single agents and in combination with cisplatin with different response rates (RR), e.g. Vinorelbine as single agent gave 24% RR in 29 patients (Steele *et al*, 2000), paclitaxel with cisplatin gave 6% RR in 18 patients (Caliandro *et al*, 2000) and gemcitabine and cisplatin gave 47% RR in 21 patients (Byrne *et al*, 1999). The survival curves in this study are very similar to our own study. Another promising drug is oxaliplatin (Ducreux *et al*, 1998) which, when combined with raltitrexed, gave a RR of 41% in 17 patients and was well tolerated as an out-patient regimen (Fizazi *et al*, 2000). The activity of single agent raltitrexed is under investigation by the EORTC and the antimetabolite MTA (multitargeted antifolate), which also gave encouraging results in the phase I study (Thodtmann *et al*, 1999), is currently in phase III testing. There are also some promising phase II data using docetaxel which gave a 23% RR in a group of 22 patients when given at a dose of 100 mg m^−2^ (Vorobiof *et al*, 2000).

Thus for the first time we are faced with a number of potentially active palliative treatments for mesothelioma but the best choice and the integration of these treatments into current management strategies awaits the initiation and completion of phase III studies. We feel the results from the study show that giving doses of SRL172 at 10^7^ bacilli per 0.1 ml dose (1 mg) IP is safe and thus this should be the dose used for phase II/III studies.
